# Validation of prognostic stroke scores—comments on “Evaluation of FRESH scores in predicting outcome and quality of life after aneurysmal subarachnoid haemorrhage in a European patient cohort”: Author’s response

**DOI:** 10.1007/s00701-024-06048-4

**Published:** 2024-04-02

**Authors:** Björn B. Hofmann, Rainer Kram, Kerim Beseoglu, Jan F. Cornelius

**Affiliations:** 1 Department of Neurosurgery, Medical Faculty and University Hospital Düsseldorf, Heinrich-Heine-University, Düsseldorf, Germany; 2 Department of Anesthesiology, Medical Faculty and University Hospital Düsseldorf, Heinrich-Heine-University, Düsseldorf, Germany

Dear Editor,

We extend our gratitude to Witsch et al., the authors of the FRESH scores [1], for their keen interest in our article titled “Evaluation of FRESH scores in predicting outcome and quality of life after aneurysmal subarachnoid haemorrhage in a European patient cohort” [2]. We value the opportunity to address their insightful remarks.

Firstly, we would like to address their characterization of our study as a validation study. While the initial intention may have been to validate the FRESH score, we must clarify that the primary objective of the performed study was to conduct an evaluation of the FRESH scores in a European cohort of patients with aneurysmal subarachnoid hemorrhage (aSAH), mainly to find out whether it is a good way of predicting QoL in our clinical practice [2]. We endeavored to articulate this clearly in our manuscript, alongside a thorough discussion of the study’s limitations and the necessity for further prospective external validation.

While our patient cohort and assessment methods may differ somewhat from those of the original FRESH score study, it is important to acknowledge that expecting perfect alignment between external populations and those used for score derivation is unrealistic. Our aim was to assess the applicability of the FRESH scores in a current clinical setting, reflecting the evolving landscape of aSAH management.

We acknowledge the concern regarding the varying observation times, which we, like Witsch et al., recognize as a significant limitation arising from the retrospective nature of our study.

It is true that in our study, 56% of patients were clipped and 44.4% were coiled [2], whereas in the original FRESH score cohort, 74% of patients with aSAH were clipped and 26% were coiled [1]. However, the distribution in our study better reflects the typical distribution observed in current patient cohorts with aSAH, particularly given the escalating prevalence of endovascular interventions in recent years [3, 4]. Additionally, we opted for the SF-36, a well-established tool for assessing quality of life, which is partially considered to have advantages over the SIP [5]. However, as both tools are validated methods for analyzing QoL, we would have expected a corresponding correlation between the FRESH score and QoL, regardless of the tool used, but we were unable to observe this in our study cohort.

In summary, our study represents a retrospective application of the FRESH scores in a genuine current European aSAH patient cohort, wherein the anticipated predictive power for outcomes, particularly quality of life, was not evident. This underscores the challenges inherent in assessing QoL as outcome variable and highlights the importance of prospective studies for the ultimate validation of predictive scores concerning QoL. It is indeed desirable to develop and validate prognostic tools such as the FRESH scores to predict QoL, alongside conventional outcome parameters, following aSAH, especially those that can be effectively applied in everyday clinical practice.

## References

[1] Witsch J et al (2016) Prognostication of long-term outcomes after subarachnoid hemorrhage: the FRESH score. Ann Neurol 80(1):46–58 27129898 10.1002/ana.24675

[2] Hofmann BB et al (2024) Evaluation of FRESH scores in predicting outcome and quality of life after aneurysmal subarachnoid haemorrhage in a European patient cohort. Acta Neurochir (Wien) 166(1):29 38261024 10.1007/s00701-024-05909-2 PMC10806023

[3] Rosenwasser RH, Chalouhi N, Tjoumakaris S, Jabbour P (2014) Open vs endovascular approach to intracranial aneurysms. Neurosurgery 61:121–129 25032540 10.1227/NEU.0000000000000377

[4] Lawton MT, Vates GE (2017) Subarachnoid hemorrhage. N Engl J Med 377(3):257–266 28723321 10.1056/NEJMcp1605827

[5] Ho AK et al (2004) Health-related quality of life in Huntington’s disease: a comparison of two generic instruments, SF-36 and SIP. Mov Disord 19(11):1341–1348 15389986 10.1002/mds.20208

